# Individualized Assessment of Exercise Capacity in Response to Acute and Long-Term Enzyme Replacement Therapy in Pediatric Pompe Disease

**DOI:** 10.3390/jpm11111105

**Published:** 2021-10-28

**Authors:** Ronen Bar-Yoseph, Galit Tal, Elena Dumin, Moneera Hanna, Gur Mainzer, Merav Zucker-Toledano, George Shallufi, Mira Jahshan, Hanna Mandel, Lea Bentur

**Affiliations:** 1 Ruth Children’s Hospital, Pediatric Pulmonary Institute, Technion Faculty of Medicine, Haifa 3109601, Israel; r_bar-yoseph@rambam.health.gov.il; 2 Metabolic Unit, Ruth Children’s Hospital, Technion Faculty of Medicine, Haifa 3109601, Israel; g_tal@rambam.health.gov.il; 3 Metabolic Laboratory Unit, Rambam Health Care Campus, Haifa 3109601, Israel; duminelena@gmail.com; 4 Ruth Children’s Hospital, Pediatric Pulmonary Institute, Haifa 3109601, Israel; m_hanna@rambam.health.gov.il (M.H.); g_shallufi@outlook.com (G.S.); 5 Pediatric Cardiology, Baruch Padeh Medical Center, Poriya 1528001, Israel; gurmainzer@gmail.com; 6 Ruth Children’s Hospital, Pediatric Cardiology Institute, Haifa 3109601, Israel; meravzt8@gmail.com; 7 Technion Faculty of Medicine, Haifa 3109601, Israel; mira.jahshan@campus.technion.ac.il (M.J.); h_mandel@rambam.health.gov.il (H.M.); 8 Department of Genetics and Metabolic Disorders, Ziv Medical Center, Safed 13100, Israel; 9 Children’s Hospital, Rambam Health Care Center, Pediatric Pulmonary Institute, Haifa 3109601, Israel

**Keywords:** infantile-onset Pompe disease (IOPD), enzyme replacement therapy (ERT), cardiopulmonary exercise testing (CPET), 6 min walking test (6MWT), oxygen uptake at the peak of exercise (peak VO_2_)

## Abstract

Background: Enzyme replacement therapy (ERT) with alglucosidase alfa improves the prospect of patients with infantile-onset Pompe disease (IOPD). However, a progressive decline has been reported. Objective quantification of the response to ERT when assessing newer strategies is warranted. Methods: This combined retrospective-prospective study assessed the acute and long-term effects of ERT on exercise in IOPD patients. Evaluation included cardiopulmonary exercise testing (CPET), 6-min walking test (6MWT), spirometry, motor function test (GMFM-88) and enzyme blood levels. Results: Thirty-four CPETs (17 pre- and 17 two days-post ERT) over variable follow-up periods were performed in four patients. Two days following ERT, blood enzyme levels increased (median, 1.22 and 10.15 μmol/L/h (*p* = 0.003)). However, FEV1, FVC and GMFM-88, the median 6MWD and the peak VO_2_ were unchanged. Long-term evaluations showed stabilization in young patients but progressive deterioration in adolescents. Clinical deterioration was associated with more pronounced deterioration in peak VO_2_ followed in the decreasing order by 6MWD, FVC and GMFM-88. Conclusions: The peak VO_2_ and 6MWD might serve as more sensitive markers to assess clinical deterioration. More studies are needed to clarify the sensitivity of the peak VO_2_ and 6MWT for quantification of individualized response. This may be important when assessing newer strategies and formulations in IOPD.

## 1. Introduction

Pompe disease is an inherited autosomal recessive glycogen storage disease caused by partial or total deficiency of acid α-glucosidase (GAA), resulting in massive accumulation of glycogen in lysosomes of different tissues. Severity of the disease and involvement of various organs are considered to be related to residual GAA activity [1]. In infantile-onset Pompe disease (IOPD), there is complete or near complete loss of GAA activity, and patients present early in life with severe hypotony, hypertrophic cardiomyopathy and early death without treatment. Patients with the late-onset disease present with progressive muscle weakness that may lead to loss of mobility and assisted ventilation, but without cardiac involvement [2]. In 2006, ERT with recombinant human GAA (Genzyme Corporation, Cambridge, MA, USA) was approved and since then, decisive modification of the course of the disease has been reported.

Clinical studies in infants have shown that ERT led to complete resolution of cardiomyopathy and improvement in skeletal muscle functions with achievement of independent walking, higher levels of physical activity and survival beyond infancy [3,4]. However, motor skills improvement in infancy is followed by a progressive decline. Different factors are considered to contribute to the clinical course [5], including age at initiation of ERT, extent of the baseline pathology, antibodies formation, cross-reactive immunologic material (CRIM) status [6], as well as explanations related to ERT distribution in skeletal muscle or other pathomechanisms, such as abnormal autophagy.

The multifactorial and heterogeneous response and the clinical deterioration on ERT suggests that enzyme replacement does not keep up with the rate of glycogen accumulation. The possibility that increasing drug delivery by either increasing dosage or frequency of administration or by means of newer formulations might halt the progression of the disease has been suggested. Common evaluation parameters include patient-reported outcome measures (PROMs), motor function scores, pulmonary function tests and walking assessments (e.g., 6 min walking test, 6MWT) for ambulatory patients [7,8,9].

Outcome parameters that may assess disease progression in newer strategies are needed. Patients’ exercise limitations contribute significantly to the patients’ perception of impaired well-being. While the focus on metabolic/functional capacity (e.g., cardiopulmonary exercise testing, CPET) in adult late-onset Pompe disease (LOPD) is growing rapidly, the data on pediatric IOPD patients are scarce [10,11]. Our aim was to provide a more objective quantification of the acute and long-term effect of ERT on exercise capacity and explore the possible relationship between the blood enzyme level and exercise capacity. Such an evaluation may help to compare disease progression with the use of newer strategies and formulations.

## 2. Materials and Methods

This was a prospective and partially retrospective case-control study assessing patients with IOPD. Diagnosis was confirmed by deficient GAA activity in cultured fibroblasts or by means of ultrahigh-performance liquid chromatography-tandem mass spectrometry (UPLC-MS/MS) in dry blood spots and by means of mutational analysis of genomic DNA. Cross-reactive immunologic material (CRIM) status was determined by means of Western blot analysis in cultured skin fibroblasts or according to the predicted GAA mutation severity.

The study was approved by the institutional review board (IRB-045-18). Written consent was obtained from the pediatric patients (assents) and from their legal guardians (the parents of the minors). Evaluation was carried out in our CPET lab, situated in a tertiary university-affiliated medical center. The inclusion criteria were as follows: Pompe disease, age of 5–18 years, replacement ERT ≥ 1 year, capable of cycling on a stationary bicycle or walking/running on a treadmill. The exclusion criteria were as follows: invasive ventilation and/or continuous oxygen dependence 24/7 or acute illness on the visit day or one day before.

### 2.1. Study Measures

Each patient underwent evaluation prior to and two days after infusion of GAA ERT (Genzyme) on multiple occasions over a variable follow-up period. Dosing was clinically adjusted and prescribed by the head of our metabolic unit. Evaluation included CPET, 6MWT, motor function score (GMFM-88) and self-collected blood samples (on a Guthrie card) for blood GAA enzyme levels.

Pre-ERT: The patients arrived at 7:00 a.m., anthropometrics and vital signs (resting heart rate, pulse oximetry and blood pressure) were collected, and a complete neuromuscular evaluation was carried out (gross motor function measure score sheet (GMFM-88), 6MWT, pre-CPET questionnaire (demographics, physical activity level, risk assessment, asthma/atopy/smoking history, family history), pulmonary function tests and CPET. Following evaluation, the patient started infusion of ERT.

Post-ERT: Two days following visit 1, a similar evaluation was performed.

#### 2.1.1. Spirometry

Spirometry was performed in accordance with the ATS/ERS (American Thoracic Society/European Respiratory Society) Task Force using a Quark PFT spirometer (Cosmed, Italy) [12]. Measurements of respiratory functions, FVC (forced vital capacity), FEV1 (forced expiratory volume in the first second), FEV1/FVC and FEF25-75 (forced expiratory flow, 25–75%) and MVV (maximum voluntary ventilation), were recorded [13].

#### 2.1.2. CPET

CPET was performed following spirometry and 15 min of rest using a Quark CPET metabolic cart (Cosmed, Rome, Italy) according to the ATS guidelines [14]. All the exercise tests were carried out by the same experienced physician (R.B.-Y.) and the same technician (H.M.) using an ergocycle, beginning with no resistance warm-up lasting 1–3 min followed by incremental increase in resistance adapted to the patient’s functional capacity according to the examiner’s free judgment and ranging from 5 to 20 Watts/minute (ramp protocol). Those patients who were unable to perform the test on a cycle ergometer were tested on a treadmill with an equivalent incremental protocol. The cardiorespiratory parameters measured included 12-lead ECG, respiration rate (RR), heart rate (HR), oxygen saturation (SpO_2_), blood pressure (BP), tidal volume (TV), minute ventilation (VE) ventilatory reserve, pulmonary oxygen uptake (VO_2_), carbon dioxide flow rejected by the ventilation system (VCO_2_), respiratory equivalents (VE/VO_2_, VE/VCO_2_), end tidal carbon dioxide (ETCO_2_), anaerobic threshold (AT), oxygen pulse (O_2_ pulse) and respiratory exchange ratio (RER). The criteria for terminating the test were as follows: inability to maintain pedaling cadence (<60 rpm) in association with subjective evidence of fatigue (sweating, hyperpnea) and one or more of the following: peak VO_2_ > 80% of the predicted value, maximum heart rate > 80% of the HR predicted (HRpred = 208 − (age × 0.7)) [15,16], RER > 1.0 or reaching the VO_2_ plateau (failure to increase oxygen uptake despite a continuous increase in work) [17]. Breathing reserve (BR) was calculated as follows: (MVV-peak VE)/MVV; low BR was defined as BR % < 15% or BR < 11 L/min [18].

#### 2.1.3. Six-Minute Walking Test

Six-minute walking test was performed according to the American Thoracic Society (ATS) guidelines [19]. SpO_2_, BP, HR and RR were evaluated pre- and post-test. As recommended, the patients were instructed to walk as far as possible along a 30-m-long flat corridor for six minutes; 6MWD was expressed in meters and the age-related percentage predicted [20,21].

#### 2.1.4. Motor Function

Motor function: Muscle strength was evaluated by a designated and experienced physiotherapist. Grading was performed according to the gross motor function measure score sheet (GMFM-88) [22]. This is a standardized observational instrument designed and validated to measure change in the gross motor function over time in children. Five dimensions are evaluated: “lying and rolling”, “sitting”, “crawling and kneeling”, “standing” and “walking, running and jumping”. The scoring ranges from 0 = does not initiate, 1 = initiates, 2 = partially completes, 3 = completes, NT = not tested.

#### 2.1.5. Evaluation of Alpha-Glucosidase Activity in Dried Blood Spots

Self-collected blood samples (on a Guthrie card) were requested. Lysosomal GAA was extracted from dried blood spots and incubated with an enzyme-specific cocktail containing the corresponding substrate and the internal standard. This enzyme cocktail was prepared using a commercially available mixture of the substrate and the internal standard at the predetermined optimized molar ratio. After incubation, the enzymatic reaction was quenched; the mixtures of the reaction products were prepared using liquid-liquid extractions and quantified simultaneously using selected ion monitoring on a UPLC-MS/MS system. Mass spectrometric analysis was performed using a tandem mass spectrometer (XEVO TQ-S MICRO, UPLC, Waters Ltd., Manchester, UK) equipped with a UPLC separation module (ACQUITY H CLASS, Waters Ltd., Manchester, UK) with an electrospray ionization source operating in the electrospray-positive mode. Quality control, data processing and analysis were performed according to the good laboratory practice. For each reaction, low (enzyme activity below the cutoff value of 2 μmol/L/h) and high (enzyme activity above the cutoff value of 100 μmol/L/h) controls from the CDC were used. The blank sample was filter paper without spotted blood.

### 2.2. Statistics

Descriptive analysis was used. The results are expressed as the median and IQR25–75. The pre-ERT parameters were compared to the post-ERT ones by means of a non-parametric paired *t*-test. Pearson’s correlation was used to assess the correlation between the parameters before and after ERT. A *p*-value lower than 0.05 was assumed as significant. The coprimary efficacy endpoints were CPET and 6MWD.

## 3. Results

Of the 10 IOPD patients followed by the metabolic unit, six patients were excluded (due to continuous mechanical ventilation, oxygen dependence and/or inability to walk/cycle). Hence, only four patients (5–11 years old) were capable of performing repeated evaluation and were included in the analysis. The patients’ characteristics are shown in Table 1. The four patients successfully performed all the measurements (spirometry, GMFM-88, 6MWT, CPET) before and two days after ERT administration on several timepoints. No adverse effects were reported during the study.

CPET: The standard Bruce ramp protocol (treadmill) was used for the younger patients (1, 2) and the standard 10 W/min ramp protocol (cycle ergometer) was used for the older patients (3, 4). The criteria for the maximum test were met by all the patients in all the tests. The duration of the test was as expected for a ramp CPET protocol (≈10 min). Exercise-induced bronchoconstriction (drop of ≥12% in FEV1) was not observed on any of the study visits. All the patients reached the target heart rate (>80% of the predicted value) at all the CPETs. No drop in oxygen saturation was observed.

### 3.1. Paired Evaluation before and Two Days after Enzyme Administration

A total of 34 (17 pre- and 17 post-ERT) comprehensive evaluations were performed in these four patients. Table 2, Table 3, Table 4 and Table 5 demonstrate the major parameters before and two days after ERT.

Analysis of the paired blood enzyme activities showed a significant increase from the median 1.22 μmol/L/h (IQR25–75, 0.83–1.43 μmol/L/h) to the median 10.15 μmol/L/h (IQR25–75, 8.7–27.5 μmol/L/h) two days after enzyme administration; *p* = 0.003.

Pulmonary function tests (FEV1, FVC) showed no significant increase two days post-ERT (FEV1 median, 82% (IQR25–75, 76–94%) and 81% of the predicted value (IQR25–75, 77–89%); FVC median, 78% (IQR25–75, 73–94%) and 78% of the predicted value (IQR25–75, 74–92%); *p* = 0.38 and *p* = 0.82, respectively). The median 6MWD was 504 m pre-ERT (IQR25–75, 446–532.5 m) and 531 m (IQR25–75, 462.5–562.5 m) post-ERT, *p* = 0.13. There was a nonsignificant increase in the peak VO_2_ (median, from 61% of the predicted value pre-ERT (IQR25–75, 53–66%) to 63% (IQR25–75, 53–66%) post-ERT; *p* = 0.063).

### 3.2. Long-Term Effect of ERT

**Figure 1:**
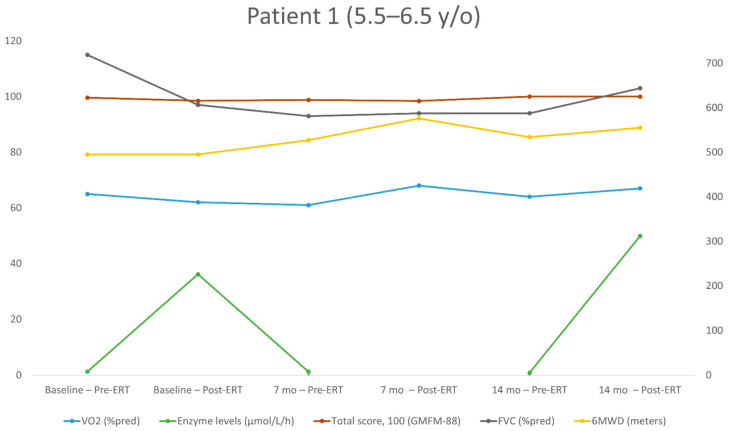
Long-term follow-up of the study parameters (patient 1). y/o = year old, VO_2_ = peak oxygen uptake at the peak of exercise; %pred = % of the predicted value; FVC = forced vital capacity; 6MWD = 6-min walking distance.

**Figure 2:**
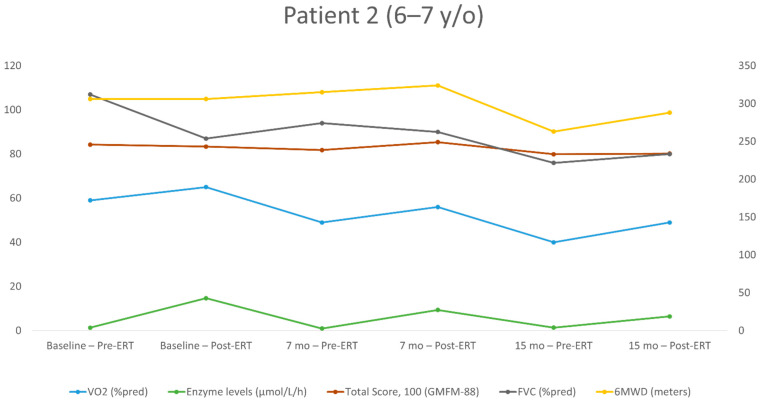
Long-term follow-up of the study parameters (patient 2). y/o = year old; VO_2_ = peak oxygen uptake at the peak of exercise; %pred = % of the predicted value; FVC = forced vital capacity; 6MWD = 6-min walking distance.

**Figure 3:**
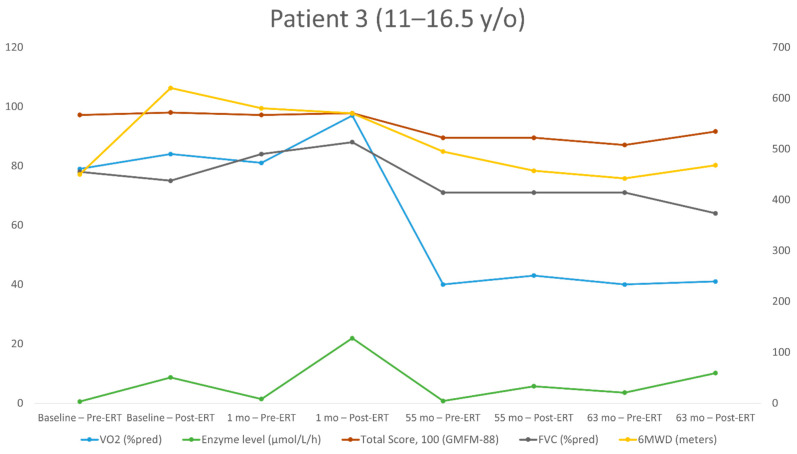
Long-term follow-up of the study parameters (patient 3). y/o = year old; VO_2_ = peak oxygen uptake at the peak of exercise; %pred = % of the predicted value; FVC = forced vital capacity; 6MWD = 6-min walking distance.

**Figure 4:**
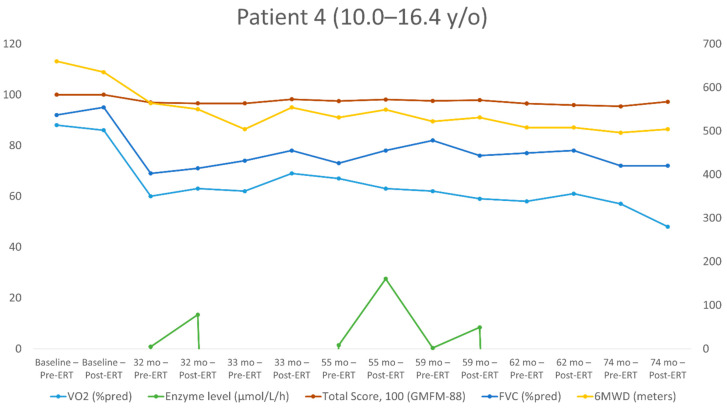
Long-term follow-up of the study parameters (patient 4). y/o = year old, VO_2_ = peak oxygen uptake at the peak of exercise; %pred = % of the predicted value; FVC = forced vital capacity; 6MWD = 6-min walking distance.

The repeated measurements for each patient over the study period are shown in Table 2, Table 3, Table 4 and Table 5. The individualized patterns of the main outcome parameters (enzyme levels, GMFM-88, 6MWT, VO_2_) are presented in Figure 1, Figure 2, Figure 3 and Figure 4.

Patient No. 1 (Table 2, Figure 1) started evaluation at the age of 5.5 years and had six evaluations (three measurements before ERT and three after). At the time of her first evaluation and throughout the study, she was seemingly healthy, with normal developmental motor skills and only minor abnormal findings on her physical examination, suggestive of myopathy. At the baseline, all the parameters evaluated were within the normal range except for the peak VO_2_. Over 14 months, FVC increased from 97% to 103% of the predicted value, GMFM-88 increased by 1.5 points (from 98.5 to 100), 6MWD increased by 60 m (from 495 to 555 m; from 104% to 108% of the predicted value) and the peak VO_2_ increased from 62% to 67% of the predicted value; from 29.0 to 30.0 mL/kg/min.

Patient No. 2 (Table 3, Figure 2) started evaluation at the age of 6.2 years and had six evaluations (three measurements before ERT and three after). This boy had a waddling gate and delayed gross motor development. He remained clinically stable during the study period. At the baseline, he had normal pulmonary function test results with reduced GMFM-88, 6MWT and peak VO_2_. Over 15 months, FVC decreased from 87% to 80% of the predicted value, GMFM-88 decreased by 3.2 points (from 83.4 to 80.2), 6MWD decreased by 18 m (from 306 to 288 m; from 59% to 52% of the predicted value) and the peak VO_2_ decreased from 65% to 49% of the predicted value; from 28.3 to 23.6 mL/kg/min.

Patient No. 3 (Table 4, Figure 3) started evaluation at the age of 11.2 years and had eight evaluations (four measurements before ERT and four after). At the time of his first assessment, this patient was seemingly asymptomatic. However, in the previous several years, there was a constant decline in motor skills and complications of peripheral neuropathy. During the last four assessments, he was able to walk only short distances and required a wheelchair for longer distances. At the baseline, he had mildly reduced pulmonary function tests, 6MWT and peak VO_2_ and normal GMFM-88. Over 63 months, FVC decreased from 75% to 64% of the predicted value, GMFM-88 decreased by 6.4 points (from 98.0 to 91.6), 6MWD decreased by 152 m (from 620 to 468 m; from 99% to 86% of the predicted value) and the peak VO_2_ decreased from 84% to 41% of the predicted value; from 28.9 to 19.4 mL/kg/min.

Patient No. 4 (Table 5, Figure 4) started evaluation at the age of 10.0 years and had 14 evaluations (seven measurements before ERT and seven after). At the time of his first assessments, this patient was also seemingly asymptomatic. However, in the previous several years, there was a slow decline in his motor skills and complications of peripheral neuropathy. He was still mobile and did not require any support. At the baseline, all the parameters evaluated were within the normal range. Over 74 months, FVC decreased from 95% to 72% of the predicted value, GMFM-88 decreased by 2.8 points (from 100 to 97.2), 6MWD decreased by 131 m (from 635 to 504 m; from 100% to 92% of the predicted value) and the peak VO_2_ decreased from 86% to 48%; from 40.0 to 19.5 mL/kg/min.

## 4. Discussion

In this study, we evaluated both acute and long-term functional capacity in response to GAA ERT in pediatric IOPD patients. Thirty-four CPET evaluations were performed (17 before and 17 two days after ERT) over variable follow-up times (14–74 months) in the patients receiving ERT since infancy. At the baseline, three of the four patients already had a decrease in oxygen uptake (peak VO_2_), while most of the other parameters were mildly reduced or unaffected. The acute response (two days after infusion of GAA ERT) showed increased blood enzyme levels but no significant changes in FEV1, FVC, GMFM-88, 6MWD or the median peak VO_2_. Long-term evaluations showed an improvement and stabilization in young patients but progressive time- and age-related clinical deterioration. The most pronounced deterioration was in the peak VO_2_, followed in the decreasing order by 6MWD, FVC and GMFM-88. This observation suggests that the peak VO_2_ and 6MWD might serve as more sensitive markers to assess clinical deterioration, and this finding may be important, especially when assessing newer strategies and formulations.

IOPD is a very rare disease [23]; of the 10 patients followed at our center, only four were able to perform repeated evaluations. All the patients were diagnosed with CRIM-positive IOPD at infancy, had cardiomyopathy at presentation and started ERT between the age of 1–7 months. ERT since infancy led to resolution of cardiomyopathy, respiratory and skeletal muscle functions improvement, achievement of independent walking and survival beyond infancy. The four patients completed their first evaluations at the prepubertal stage. In our study, the older patients received GAA ERT for a period of 16 years, the younger ones—for 6 years.

A high degree of variability was observed among the patients in their responses to treatment. A recent report described long-term outcomes in 14 IOPD CRIM-positive patients and showed that ERT maintained the respiratory function in a subgroup of patients, whereas others had a steady decline [7].

The acute and long-term follow-up of patients on ERT is necessary to better understand the variable response and the challenges that hinder its effectiveness and explore the unmet needs.

Objective quantification of the response to ERT, especially when assessing newer strategies and formulations, is warranted. Exercise capacity significantly affects Pompe disease patients’ clinical picture and quality of life. CPET outcome may potentially be research and clinical functional assessment of ERT.

The reported data on the acute and long-term effects of ERT on CPET parameters is limited, especially in IOPD. The safety of exercise and CPET was demonstrated in LOPD [10,24], and we recently demonstrated the feasibility and safety of CPET in five pediatric Pompe disease patients [11].

Baseline parameters: At the first assessment, only one patient had mildly reduced FCV, one had reduced motor score and one had reduced 6MWD; this may reflect the change in the natural course of IOPD under early administration of ERT. A recent multicenter study from India (with limited access to ERT) [25] showed improved outcomes and survival in patients receiving ERT compared to those not receiving ERT. However, three of the four patients already had a decrease in oxygen uptake (peak VO_2_), which represents one of the main indices of exercise capacity. This may imply that the peripheral muscles may be more prominent in determining the peak VO_2_ decrease than the cardiorespiratory component in IOPD; the peak VO_2_ might be more sensitive than the pulmonary function, GMFM-88 and 6MWT when evaluating clinical status in IOPD.

Acute response to ERT: In clinical practice, Pompe disease patients treated for a long time with ERT often report a subjective improvement of general well-being and reduced fatigability following administration, with a decline toward the end of the two-week interval between infusions.

When we assessed the response two days after ERT, all the patients reported subjective improvement, which correlates with increased enzyme activity measured in DBS. However, this improvement was not supported by either motor assessment or pulmonary function tests. There was a nonsignificant increase in 6MWD and the peak VO_2_. We are aware of one similar study assessing the acute effect (one day before and after ERT) on CPET parameters and blood enzyme levels in 11 LOPD patients [26]. Similarly, to this study, no significant differences in the variables related to exercise tolerance were found despite a significant increase in GAA activity in peripheral lymphocytes.

This may imply that these parameters are not sufficiently sensitive to assess individualized acute response to ERT. The small sample size may have resulted in a type 2 error. Alternatively, the increase in blood enzyme levels following therapy may have affected other unmeasured parameters (e.g., the quality-of-life questionnaire, smart watches assessing the activity).

Long-term response to ERT: Two small case series of LOPD assessed the effect of ERT on repeated CPET measures [10,24]. Marzorati et al. demonstrated the positive effect of ERT on some physiological variables associated with exercise tolerance in four patients with LOPD after one year of therapy [24]. Crescimanno et al. evaluated eight LOPD patients by means of CPETs, but only four of them (one—naïve to ERT) were followed up after 36 months of treatment.

Three patients showed a reduced exercise capacity as evaluated by peak oxygen uptake (VO_2_) measured at the CPET and 6MWT at the baseline. At the follow-up, 9.5% improvement in oxygen uptake and 5.6% (25 m) improvement in 6MWD was reported. The difference may be related to different type of disease (late vs. infantile onset), duration and dose of ERT used, response variability and sample size [10]. A case study of a 50-year-old female with Pompe disease showed improvement in CPETs after one month of ERT and no further changes following 24 months of ERT [27]. We are unaware of studies repeating CPET measurements over a long-term follow-up period in IOPD. In our study, the two younger patients (patients 1 and 2) remained stable over 14–15 months of follow-up. The two older patients (patients 3 and 4) showed relative stability at their first evaluations. However, when reassessed during puberty and at a post-pubertal stage, they had started to deteriorate. Patients 2–4 are CRIM positive and did not receive any immune suppressant protocol. Patient 1 is compound heterozygote including a severe mutation R854X and the CRIM positive L355P. She received a single course of three consecutive daily methotrexate injections; first injection was given just before the very first Myozyme infusion. None of the patients had significant neutralizing antibody levels. Two patients had zero levels (#3, #4), one patient (#1) had antibody titer of 1600 and one patient (#2) had 1800. Levels of ≥6400 are considered significant [28], therefore, the waning efficacy cannot be attributed to antibodies formation.

The active functional evaluations (e.g., 6MWT, CPET) seemed to better correlate with the reported clinical deterioration. The 6MWT is a relatively common method to assess progression in Pompe disease and other neuromuscular disorders. However, VO_2_ peak was affected more than 6MWT, suggesting that it is a more sensitive parameter to evaluate individualized response.

Similarly, Sechi et al. suggested that VO_2_ peak might be more sensitive than 6MWT in estimating exercise tolerance in LOPD. The decline in exercise capacity was not explained by overt cardiac or respiratory limitations and probably is related to muscle weakness and deconditioning. Motor assessment by our experienced physiotherapist showed a lower correlation to clinical deterioration, even when specific functional dimensions (e.g., walking, running & jumping) were measured (Table 2, Table 3, Table 4 and Table 5). This suggests that other measures of muscles functions (e.g., MRI, biopsy) should be used.

Indeed, ERT does not cure the disease and the majority of IOPD infants develop progressive myopathy in subsequent years, despite very early initiation of treatment. The most apparent limitation of ERT is the poor response of skeletal muscles with accumulation of glycogen-filled autophagosomes [29]. In LOPD, ERT resulted in stabilization or slowing of progression of the disease. A large double-blind placebo-controlled study carried out in 90 adult patients demonstrated improved walking distance and stabilization of the pulmonary function over an 18-month period [2]; the largest response was seen during the first 2–3 years of treatment [30].

The clinical deterioration observed in IOPD patients on ERT suggests enzyme replacement does not keep up with the rate of glycogen accumulation, and significant unmet medical needs remain. Despite long-term and regular administration of ERT, there is histological evidence of pronounced autophagic vacuoles in skeletal muscle specimens but only a small amount of glycogen storage in lysosomes [29]. This finding could explain in part the progressively reduced effect of ERT, resulting in motor deterioration in these patients [29]. The possibility that increasing drug delivery by either increasing dosage or frequency of administration might halt the progression of the disease was, therefore, suggested.

In our study, two days following ERT, significantly increased blood enzyme levels were revealed (median, 1.22 μmol/L/h and 10.15 μmol/L/h; *p* = 0.003). Enzyme levels in response to ERT are rarely reported [26]. It should be acknowledged that GAA activity measured in peripheral blood does not necessarily reflect the levels of activity achieved in skeletal muscles. However, determination of GAA activity in muscle tissues would require a muscle biopsy, which was not carried out in this study for ethical reasons.

Few studies have reported on the effect of increasing ERT dosage on the efficacy of treatment [29]. One could speculate that higher enzyme levels may escape autophagy. The preliminary data of a recent study which compared the safety and efficacy of treatment with 40 mg/kg/week to that of 20 mg/kg/every other week (EOW) showed that a higher dose leads to improvement in Pompe disease patients [31]. In this study, the dose was prescribed and changed by the head of the metabolic unit. The initial ERT dosage in patients 3 and 4 was 20 mg/kg EOW as recommended by results of the studies performed in 2003–2006 [4]. During the recent years, their dose was increased to 40 mg/kg EOW or 20 mg/kg EW (patient 3) due to the policy change in Israel based on results of several studies which recommended high-dose ERT, 40 mg/kg EOW [32,33]. Patients 1 and 2 had been receiving this higher dose since starting therapy. The low number of patients involved in the study, the different ERT dosages at variable times and the low number of self-collected blood samples precluded further analysis.

Limitations: The major limitation, as in other orphan diseases, is the small sample size. Some of the measurements were performed for clinical purposes before the study was designed, therefore the study is partially retrospective. All the patients started ERT in infancy as clinically indicated, however, with different doses and frequencies. We did not have baseline exercise capacity before GAA ERT initiation. Therefore, the effect of ERT in naïve IOPD could not be evaluated. Inspiratory/expiratory muscle strength (MIP, MEP) tests and health-related quality of life were not assessed. GAA blood enzyme levels were not collected at each visit, hence the response to different doses could not be assessed.

## 5. Conclusions

Individualized assessment of the peak VO_2_ by means of CPETs may provide a more sensitive assessment of clinical severity and long-term response to treatment.

CPET parameters may be less sensitive to assess the acute effect of ERT in non-naïve ERT patients; 6MWT may provide helpful information in regions with limited access to CPET; larger, long-term prospective studies are needed to clarify the sensitivity of the peak VO_2_ and 6MWT for quantification of individualized response and the possible relation to blood levels. This may aid in evaluation of different ERT dosages and newer formulations.

## Figures and Tables

**Table 1:** Demographics and characteristics of the study population at first evaluation.

	Patient 1	Patient 2	Patient 3	Patient 4
**Age (years)/sex**	5.5/Female	6/Male	11/Male	10/Male
**Study duration (years)**	1.2	1.3	5.3	6
**Height (cm)**	112.5	113	139	145
**Weight (kg)**	21.2	26.5	37.2	44.1
**BMI (%)**	16.8 (83)	20.8 (99)	19.3 (78)	21 (92)
**GAA mutation**	R854X/L355P	L355P/D404N	L355P/L355P	L355P/D404N
**CRIM status**	Positive	Positive	Positive	Positive
**Ethnicity**	Muslim	Druze	Druze	Druze
**Onset**	Infantile	Infantile	Infantile	Infantile
**ERT onset age (months)**	5	3	7	1
**Cardiomyopathy at infancy**	Yes	Yes	Yes	Yes
**Current echo**	Normal	Normal	Normal	Normal
**Drop foot**	No	Yes	Yes	Yes
**Gastrostomy**	No	Yes	No	No

Note: BMI = body mass index; GAA = acid α-glucosidase; CRIM = cross-reactive immunological, ERT = enzyme replacement therapy.

**Table 2:** Patient 1.

	First Evaluation		Second Evaluation (+7 Months)		Third Evaluation (+14 Months)	
	Post-ERT	Pre-ERT	Post-ERT	Pre-ERT	Post-ERT	Pre-ERT
**Age at test (years)**	5.5	5.5	6.2	6.2	6.7	6.7
**Height (cm)**	112.5	112.5	116.5	116.5	119	119
**BMI percentile**	83	83	73	73	84	84
**ERT dose (mg/kg)**	40/EOW	40/EOW	40/EOW	40/EOW	40/EOW	40/EOW
**Enzyme level (μmol/L/h)**	34.85	1.22	N/A	1.24	49.87	0.83
**FEV1 (L/s)**	1.12	1.07	1.22	1.17	1.4	1.29
**FEV1 (% of the predicted value)**	99	94	100	96	109	101
**FVC (L)**	1.19	1.42	1.26	1.25	1.46	1.34
**FVC (% of the predicted value)**	97	115	94	93	103	94
**Peak HR (bpm)**	198	197	199	195	208	199
**Absolute peak VO_2_ (mL/min)**	615	647	698	626	731	695
**Specific peak VO_2_ (mL/kg/min)**	29	30.5	31.6	28.3	30	28.5
**Peak VO_2_ (% of the predicted value)**	62	65	68	61	67	64
**VE/VCO_2_ slope**	38.7	37.8	41.7	32	42.4	33.5
**O_2_ pulse (% of the predicted value)**	64	68	70	64	65	65
**Breathing reserve**	L	N	L	N	L	N
**6MWD (meters)**	495	495	576	527	555	534
**Walking %, running (%), jumping (%) GMFM-88)**	100	100	100	100	100	100
**Total score (%) (GMFM-88)**	98.5	99.6	98.4	98.8	100	100

Note: Legends to Table 2, Table 3, Table 4 and Table 5: BMI = body mass index, ERT = enzyme replacement therapy; EOW = every other week; FEV1 = forced expiratory volume in the first second; FVC = forced vital capacity; HR = heart rate; Peak VO_2_ = oxygen uptake at the peak of exercise; VE = minute ventilation; VCO_2_ = carbon dioxide production; N = normal; 6MWD = 6-min walking distance; GMFM-88 = motor assessment.

**Table 3:** Patient 2.

	First Evaluation		Second Evaluation (+7 Months)		Third Evaluation (+15 Months)	
	Post-ERT	Pre-ERT	Post-ERT	Pre-ERT	Post-ERT	Pre-ERT
**Age at test (years)**	6.2	6.2	6.9	6.9	7.5	7.5
**Height (cm)**	113	113	118	118	123	123
**BMI percentile**	99	99	97	97	97	97
**ERT dose (mg/kg)**	20/EOW	20/EOW	40/EOW	40/EOW	40/EOW	40/EOW
**Enzyme level (μmol/L/h)**	14.68	1.34	9.35	0.95	6.45	1.35
**FEV1 (L/s)**	1.03	1.29	1.16	1.2	1.12	1.06
**FEV1 (% of the predicted value)**	87	104	88	91	77	73
**FVC (L)**	1.15	1.47	1.33	1.38	1.32	1.24
**FVC (% of the predicted value)**	87	107	90	94	80	76
**Peak HR (bpm)**	202	204	190	172	170	185
**Absolute peak VO_2_ (mL/min)**	751	721	726	644	736	590
**Specific peak VO_2_ (mL/kg/min)**	28.3	27.2	25.9	23	23.6	18.9
**Peak VO_2_ (% of the predicted value)**	65	59	56	49	49	40
**VE/VCO_2_ slope**	39.9	32.3	40	42.4	38	43.2
**O_2_ pulse (% of the predicted value)**	65	59	60	58	59	43
**Breathing reserve**	L	N	N	N	N	N
**6MWD (meters)**	306	306	324	315	288	263
**Walking (%), running (%), jumping (%) (GMFM-88)**	54.1	58.3	61.1	54.1	50	50
**Total score (%) (GMFM-88)**	83.4	84.3	85.4	81.8	80.2	79.88

**Table 4:** Patient 3.

	First Evaluation		Second Evaluation (+1 Month)		Third Evaluation (+55 Months)		Fourth Evaluation (+63 Months)	
	Post-ERT	Pre-ERT	Post-ERT	Pre-ERT	Post-ERT	Pre-ERT	Post-ERT	Pre-ERT
**Age at test (years)**	11.2	11.2	11.3	11.3	15.9	15.9	16.5	16.5
**Height (cm)**	139	139	139	139	165	165	166	166
**BMI percentile**	78	78	78	78	65	65	57	57
**ERT dose (mg/kg)**	20/EOW	20/EOW	40/EOW	40/EOW	20/W	20/W	20/W	20/W
**Enzyme level (μmol/L/h)**	8.7	0.55	9.35	2	N/A	2.85	10.15	3.55
**FEV1 (L/s)**	1.64	1.7	1.8	1.78	2.9	2.93	2.77	2.84
**FEV1 (% of the predicted value)**	81	85	89	88	80	80	73	75
**FVC (L)**	1.72	1.78	2.03	1.93	2.96	2.98	2.8	3.08
**FVC (% of the predicted value)**	75	78	88	84	71	71	64	71
**Peak HR (bpm)**	172	166	189	166	175	130	168	157
**Absolute peak VO_2_ (mL/min)**	1400	1308	1610	1346	1197	1117	1147	1119
**Specific peak VO_2_ (mL/kg/min)**	37.6	35.2	43.3	36.2	20.3	18.9	19.4	19
**Peak VO_2_ (% of the predicted value)**	84	79	97	81	43	40	41	40
**VE/VCO_2_ slope**	37.9	24.6	41.1	30.4	36.7	23.4	32.7	32.5
**O_2_ pulse (% of the predicted value)**	8.4	7.9	8.5	8.1	6.8	8.6	6.8	7.1
**Breathing reserve**	N	N	L	N	N	N	N	N
**6MWD (meters)**	620	450	570	580	457	495	468	442
**Walking (%), running (%), jumping (%) (GMFM-88)**	98.6	97.2	100	97	77.7	77.7	70.8	65.2
**Total score (%) (GMFM-88)**	98	97.2	97.8	97.2	89.5	89.5	91.6	87.1

**Table 5:** Patient 4.

	First Evaluation		Second Evaluation (+32 Months)		Third Evaluation (+33 Months)		Fourth Evaluation (+55 Months)		Fifth Evaluation (+59 Months)		Sixth Evaluation (+62 Months)		Seventh Evaluation (+74 Months)	
	Post-ERT	Pre-ERT	Post-ERT	Pre-ERT	Post-ERT	Pre-ERT	Post-ERT	Pre-ERT	Post-ERT	Pre-ERT	Post-ERT	Pre-ERT	Post-ERT	Pre-ERT
**Age at test (years)**	10	10	12.8	12.8	12.9	12.9	14.7	14.7	15.1	15.1	15.4	15.4	16.4	16.4
**Height (cm)**	145	145	167	167	167	167	177	177	177	178	178	178	186	186
**BMI percentiles**	92	92	94	92	83	83	97	97	98	98	98	98	95	95
**ERT dose (mg/kg) EOW**	20	20	20	20	40	40	40	40	20	20	40	40	40	40
**Enzyme level (μmol/L/h)**	N/A	N/A	13.4	0.85	N/A	N/A	27.5	1.43	8.45	0.34	N/A	N/A	N/A	N/A
**FEV1 (L/s)**	1.99	2.01	2.51	2.29	2.74	2.62	3.09	3.12	3.3	3.41	3.39	3.23	3.45	3.45
**FEV1 (% of the predicted value)**	89	90	74	67	80	77	77	78	80	82	81	77	74	74
**FVC (L)**	2.44	2.38	2.82	2.73	3.1	2.95	3.69	3.44	3.65	4.01	3.85	3.8	3.94	3.95
**FVC (% of the predicted value)**	95	92	71	69	78	74	78	73	76	82	78	77	72	72
**Peak HR (bpm)**	190	186	206	200	197	198	203	205	207	204	201	191	198	201
**Absolute peak VO_2_ (mL/min)**	1764	1733	2000	1908	2165	1950	2320	2440	2151	2274	2261	2134	1838	2163
**Specific peak VO_2_ (mL/kg/min)**	40	40.2	30.3	28.9	33.31	30	26.4	27.7	22.96	23.99	23.93	22.58	19.5	23.01
**Peak VO_2_ (% of the predicted value)**	86	88	63	60	69	62	63	67	59	62	61	58	48	57
**VE/VCO_2_ slope**	36	31.1	30.5	29.5	26.8	28.5	29.3	27.3	31.8	30.6	28.8	30	27.9	28.4
**O_2_ pulse (% of the predicted value)**	91	95	61	60	70	63	69	71	56	60	60	60	48	56
**Breathing reserve**	N	N	N	N	N	N	N	N	N	N	N	N	N	N
**6MWD (meters)**	635	660	550	564	554	504	549	531	531	522	508	508	504	496
**Walking (%), running (%), jumping (%) (GMFM-88)**	100	100	95.8	94.4	98.6	97.2	98.6	93	94.4	93	90.2	90.2	91.6	87.5
**Total Score %-GMFM-88**	100	100	96.58	96.9	98.2	96.58	98.1	97.5	97.84	97.56	95.9	96.5	97.2	95.4

## Data Availability

The data presented in this study are available from the corresponding author upon reasonable request.
